# Society Notes

**Published:** 1898-02

**Authors:** 


### SOCIETY NEWS.

THE next meeting of the Missouri Valley Veterinary Medical Association will be held in Kansas City, Wednesday, February 9th, and the Secretary announces a strong programme.

THE annual meeting of the Wisconsin Society of Veterinary Graduates will be held at the Assembly Chambers, Madison, on Friday, February 25th, at 1.30 P.M. The programme is a very interesting one.

AT their fifteenth annual meeting the Ohio State Veterinary Medical Association elected the following officers: President, Dr. Walter Shaw, Dayton; First Vice-President, Dr. W. J. Torrance, Cleveland; Second Vice-President, Dr. L. W. Carl, Columbus; Third Vice-President, Dr. S. D. Meyers, Wilmington; Secretary, Dr. W. H. Gribble, Washington Court House; Treasurer, T. B. Hillock, Columbus.

THE Executive Committee of the Alumni Association of the American Veterinary College will meet in the College building on February 10th, at 3 P.M., for the purpose of transacting such business as is connected with the graduation of the class of '98, by direction of Chairman Wm. Herbert Lowe, of Paterson, N. J.

THE annual meeting of the Keystone State organization will be held in Philadelphia on March 8 and 9, 1898. The great interest this meeting always awakens will be maintained this year by an unexceptionable programme of great merit. The most important committee reports will be made at this meeting, and an unusual interest all over the State is being centred in the coming gathering.

THE New York County Veterinary Medical Association will be entertained by Dr. Herbert Neher at their February meeting by an illustrated

(microscopically) discourse on developing bone, showing ossification zones; sections of hard bones, exhibiting the lacunæ, canaliculi, cartilages, etc.; sections of fœtal humerus of a cat, showing bone development; section of decalcified bone, showing minute anatomy of the same.

Dr. Oscar Busener, graduate of the American Veterinary College, class of '91, died recently at his home in Jeffersonville, New York, from tuberculosis.

